# Usefulness of fluorescence visualization-guided surgery for early-stage tongue squamous cell carcinoma compared to iodine vital staining

**DOI:** 10.1007/s10147-020-01710-0

**Published:** 2020-05-25

**Authors:** Yusuke Ikeda, Taiki Suzuki, Hirokazu Saitou, Satoru Ogane, Kazuhiko Hashimoto, Nobuo Takano, Takeshi Nomura

**Affiliations:** 1 Department of Oral Medicine, Oral and Maxillofacial Surgery, Tokyo Dental College, Ichikawa General Hospital, 5-11-13 Sugano, Ichikawa, Chiba 272-8513 Japan; 2 Oral Cancer Center, Tokyo Dental College, Ichikawa General Hospital, 5-11-13 Sugano, Ichikawa, Chiba 272-8513 Japan; 3 Division of Surgical Pathology, Clinical Laboratory, Ichikawa General Hospital, Tokyo Dental College, 5-11-13 Sugano, Ichikawa, Chiba 272-8513 Japan

**Keywords:** Fluorescence visualization, FV-guided surgery, Iodine vital staining, OSCC, Tongue cancer, Surgical resection

## Abstract

**Background:**

In the most cases of oral squamous cell carcinoma (OSCC), oral epithelial dysplasia (OED) is found adjacent to the primary tumor. The delineation of surgical margins for OSCC is critical to minimize the risk for local recurrence. The aim of this study is to demonstrate that the fluorescence visualization (FV)- device can delineated the lesion visualizes OED of adjacent primary tumors by histopathologically comparison to conventional iodine vital staining.

**Material and methods:**

The study involved 40 patients with superficial tongue squamous cell carcinoma treated from July 2016 to July 2018 at the Oral Cancer Center, Tokyo Dental College.

**Results:**

Cytokeratin 13 (CK13) expression rate in the area of fluorescence visualization loss (FVL) was significantly lower than that in the area of fluorescence visualization retention (FVR). In addition, CK17, Ki-67, and p53 expression rates were significantly higher in FVL than FVR. There was no significant difference in the delineation rate or area between FVL and iodine-unstained area. High-grade dysplasia was observed most frequently at the FV and iodine-unstained boundary, but no significant pathological differences were found.

**Conclusion:**

We strongly suggest the FV-guided surgery is a useful method for accurate resection in early-stage tongue squamous cell carcinoma.

## Introduction

Oral squamous cell carcinoma (OSCC) is the cancer with the sixth highest mortality rate worldwide, and the survival rate is low. The five-year survival rates for stage 1 tongue cancer is 80%, while the five-year survival rates for cancers of oral cavities are around 50%. This is largely reflected by the fact that many cases are in advanced stages at the time of detection. [1] The conventional treatment for early OSCC is surgical resection or radiation therapy based on the National Comprehensive Cancer Network (NCCN) guidelines [2]. Surgical resection is given priority because it is generally regarded as effective [3]. Surgeons frequently place the surgical margin at 1.5–2.0 cm from the primary tumor based on a visibly abnormal field and palpable area of induration according to the NCCN guidelines [2]. However, oral epithelial dysplasia (OED) adjacent to OSCC is difficult to discriminate. Therefore, determination of the surgical margin requires more objective evaluation. Most cases of OSCC undergo multistep carcinogenesis. Abnormal fields of the surgical margin occur due to accumulated genetic changes and histological development [4]. Thus, the surgical margin is an important therapeutic issue when treating oral cancer because a residual abnormal field results in the development of a second primary tumor.

We have identified the abnormal field using conventional methods. Toluidine blue and iodine vital staining are representative established methods [5]. Surgical resection using iodine vital staining is particularly effective for early OSCC because it allows for visualization of the boundary of OED [6]. Normal non-keratinized epithelium stains well with iodine solution; however, abnormal fields with various types of dysplasia do not stain well with iodine, exhibiting iodine-unstained (IUS) areas because of differences in the glycogen content of the cytoplasm [7, 8]. Thus, iodine staining allows for establishment of the surgical margin of OSCC, leading to an improved prognosis [9]. However, iodine vital staining has some limitations; for example, the thick oral mucosa of keratinizing layers, such as the normal gingival and hard palate contain low glycogen, and the orthokeratinized epithelium fails to take up iodine [6]. Additionally, iodine sometimes induces adverse reactions [10]. We, therefore, hope to develop new approaches as alternatives to iodine vital staining that allow for easy management of abnormalities.

The Fluorescence Visualization (FV)- device (VELscope; LED Medical Diagnostics, Inc., Vancouver, Canada) is a simple hand-held instrument. The FV- device allows for direct visualization of the abnormal field by tissue autofluorescence [11–14]. This device uses a blue light in the range of 400–460 nm, and a selective filter in the eyepiece allows the viewer to directly visualize the pale green color caused by the implicated for example collagen matrix or flavin adenine dinucleotide [12, 14, 15]. This pale green autofluorescence given off by normal tissue is called FV retention (FVR). Conversely, abnormal fields show decreased autofluorescence and appear as dark areas in contrast to the green surrounding normal tissue. Such dark areas exhibit FV loss (FVL).

The aim of this study is to demonstrate that the FV- device can delineated the lesion visualizes OED of adjacent primary tumors histopathologically compare to conventional iodine vital staining.

## Patients and methods

### Patients

This study included 40 patients with early superficial tongue squamous cell carcinoma diagnosed pathologically and surgically resected from August 2015 to August 2018 at the Oral Cancer Center, Tokyo Dental College. The patients consist of 26 men and 14 women with an average age of 60.0 years (95% confidence interval, 54.3–65.6). In this study, we defined early superficial tongue squamous cell carcinoma as a tumor of 4 cm in size (T1–T2) with no invasion into the muscle layers and no regional lymph node metastasis (N0) (Union for International Cancer Control 7th edition). No lesions were detectable by contrast computed tomography or magnetic resonance imaging. All patients underwent examinations by both FV and iodine vital staining, and all underwent partial glossectomy. The surgical margin was extended an additional 5 mm beyond the lesion boundary as guided by the FVL.

This was a prospective study. Written informed consent was obtained, and ethical clearance was provided by the Ethical Committee of Ichikawa General Hospital, Tokyo Dental College, Japan (approval No. I 17-80).

### FV-guided surgery

**Fig. 1:**
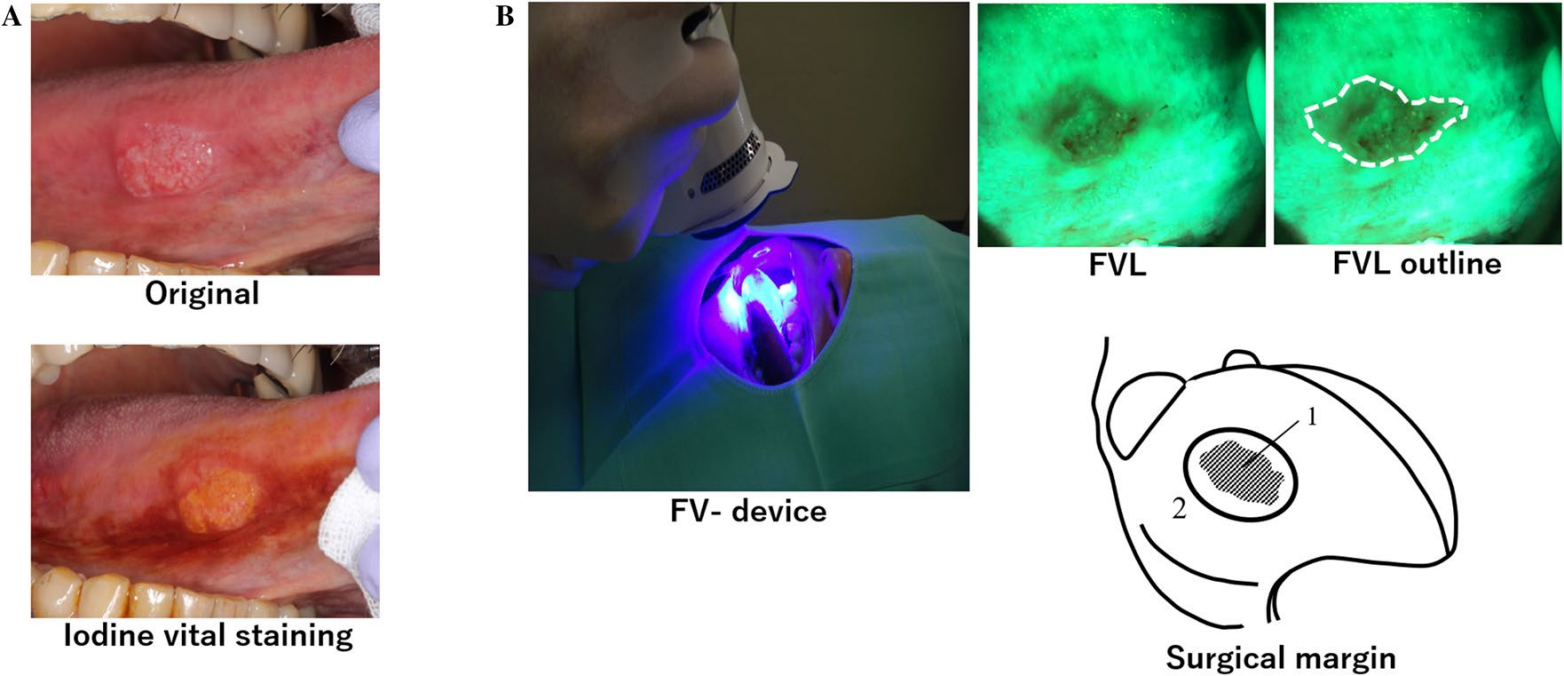
Initial assessment and measurement under white light of early tongue squamous cell carcinoma. We used to Iodine vital staining method for delineation of the iodine-unstained area as borders of dysplastic epithelium adjacent to the tongue squamous cell carcinoma (**a**). Assessment of field using FV- device. FVL is outlined in green in the dark. Demarcation of FVL boundaries. Schema showing OED adjacent to tongue squamous cell carcinoma as fluorescence visualization loss (FVL). 1. Tumor with FVL. 2, Surgical margin (**b**)

**Fig. 2:**
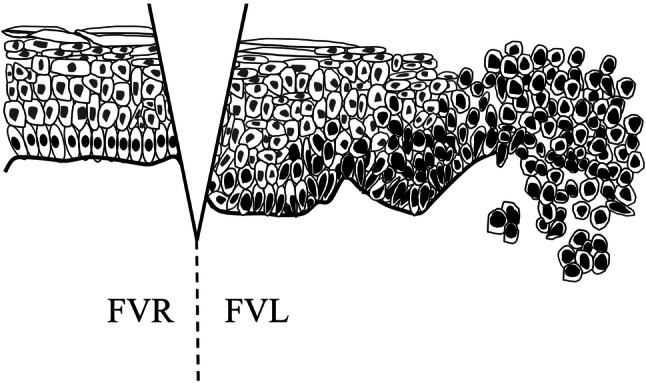
Schema showing the cutting point. We set the cutting point at the FVL and FVR boundary to prepare the pathological specimen

Observation of tissue autofluorescence was performed with a FV device. We resected the tumors with a scalpel in accordance with the FVL and IUS areas in all cases. All procedures were performed with the patients under general anesthesia, and conditions were standardized as much as possible. Step 1, initial assessment and measurement under white light of early tongue squamous cell carcinoma (Fig. 1-a). Step 2, perform iodine vital staining by Lugol’s iodine solution (aqueous iodine solution) was performed and measure the dimensions (long and short diameter) of the boundary between IUS (Fig. 1-a). Step 3, the FV device was used to illuminate the lesions and then mark (with a crystal violet) and measure the dimensions (long and short diameter) of the boundary between FVL and FVR (Fig. 1-b). Step 4, we resected tumor as guided by the FVL based on the guidelines established by the Japanese Society of Oral Oncology [16]. Step 5, after resection, the FVL boundary was marked with a scalpel. Under microscopic observation, the cut mark was used as the indicator of the FVL boundary (Fig. 2). IUS area considered as PAS negative area.

### Evaluation of malignant or premalignant biomarkers by immunohistochemistry

Forty specimens were obtained from 40 patients. Immunohistochemical staining was performed using an automatic immunohistochemistry analyzer (Roche Diagnostics, Basel, Switzerland) at room temperature. Sections were cut and placed on clean silane-coated glass slides and fixed with 10% paraformaldehyde. Four mouse monoclonal antibodies to cytokeratin 13 (CK13) (× 200 dilution; DE-K13; Dako, Glostrup, Denmark), CK17 (× 40 dilution; E3; Dako), Ki-67 (× 200 dilution; MIB-1; Dako), and p53 (no dilution; Histofine; Nichirei Corp., Tokyo, Japan) were used to determine immunohistochemical expression for FVL and FVR.

**Fig. 3:**
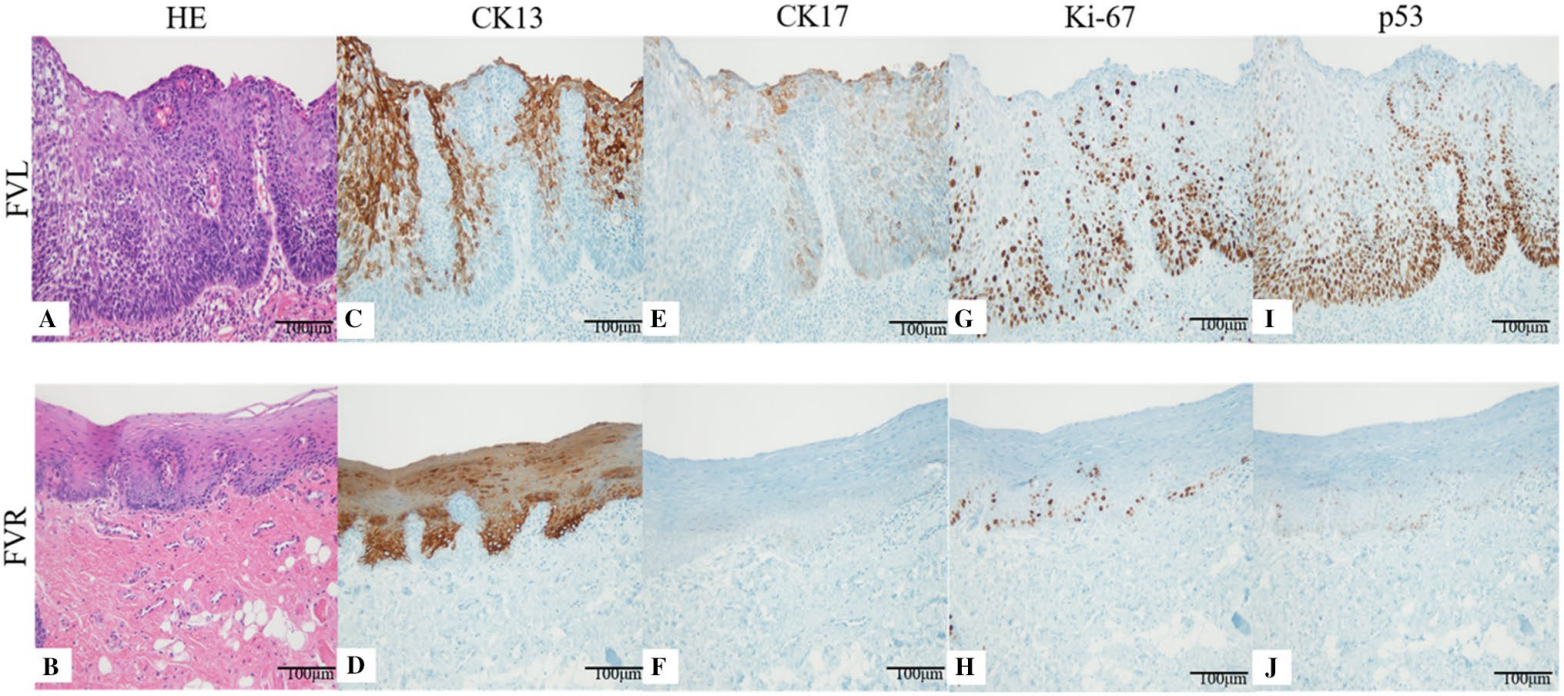
The labeling index is given as the positive cell ratios, calculated from the percentage of CK17- and CK13-positive cells or Ki-67- and p53-positive nuclei. Approximately 100–500 cells were examined in each field (× 200). **a**, **b** In the fluorescence visualization loss (FVL) area, loss of polarity of basal cells and abnormal variation in nuclear size and shape were observed. Changes were present in the upper third of the epithelium. Meanwhile, normal architecture and cytology were observed in the fluorescence visualization retention (FVR) area. **c**, **d** CK13 immunoreactivity was observed in the upper layers. The FVL showed lower expression of CK13 than the FVR. **e**, **f** CK17 immunoreactivity was observed in the upper layers and part of the lower layers. CK17 expression was rare in FVR compared with FVL. **g**, **h** Ki-67 immunoreactivity was observed in the basal and parabasal cell layers. Higher expression of Ki-67 was found in FVL than FVR. **i**, **j** p53 immunoreactivity was also observed in the basal and parabasal cell layers. Higher expression of p53 was found in FVL than FVR

The results were analyzed with optical cell counting and ImageJ 1.52a cell counter software (National Institutes of Health, Bethesda, MD, USA). The labeling index is given as an expression rate, and was calculated from the rate of CK13- and CK17-positive cells or Ki-67- and p53-positive nuclei. In total, 100–500 epithelial cells were examined in every three fields (× 200) [17] (Fig. 3).

### Comparison between FV device and iodine vital staining method

According to the iodine vital staining method, the IUS area is periodic acid-Schiff (PAS)-negative because the glycogen content is absent or lower than that of the normal epithelium [18]. Therefore, the pathological findings can be distinguished by the difference in the cytoplasmic glycogen content. We performed PAS staining (Muto Pure Chemicals, Tokyo, Japan) with an automated slide strainer (Tissue-Tek Prisma JOD; Sakura Finetek, Tokyo, Japan). PAS negativity indicated an IUS area, therefore, we compared the difference between PAS-negative areas and FVL areas.

#### Expression of PAS-positive cells in FVL area

We evaluated the glycogen content in keratinized epithelium of the FVL area by PAS staining. To examine the PAS-positive cell distribution and PAS-positive ratio, micrographs of each section were taken at a magnification of × 400 for every three foci of FVL and FVR. We calculated the labeling index using a previously described method [19]. Lines crossing at a right angle to the surface of the basal layer of the epithelium were drawn on the micrographs. PAS-positive cells touching this line were counted to calculate the number of PAS-positive cells. The expression rate of PAS staining was calculated as the number of PAS-positive cells divided by all cells touching that line [19].

#### OED in lesion boundary

We compared the pathological findings between the FVL and IUS areas at both ends of the lesion boundaries. These specimens were observed in every four foci at the boundaries. OED was classified according to the World Health Organization classification (2017) and divided into low-grade dysplasia and high-grade dysplasia using a binary system [20] (Fig. 3).

#### Comparison of delineation areas and effective rates between FV- device and iodine vital staining method

At first, we compared effective rates between the FV device and iodine vital staining method. We then measured the lesion delineation areas of the FVL and IUS in the same cases. The delineated areas were assumed to be the approximate values of the ellipses in the range of the FVL and IUS area. Calculation of these areas was performed as follows:

$${\text{Ellipse equation}}\, = \,\left( \pi \right)\, \times \,\left( {\text{half of long diameter}} \right)\, \times \,({\text{half of short diameter}}).$$

### Statistical analysis

The expression rate in immunohistochemical staining and pathological findings between the FVL and IUS lesion boundary were analyzed by the Mann–Whitney *U* test. The difference in the lesion delineation rate between the iodine vital staining method and FV device was statistically analyzed by Fisher’s exact test. The correlation between the FVL and IUS lesion delineation area were analyzed by Pearson’s product–moment correlation. A *p *value of < 0.01 was considered statistically significant. All statistical analyses were performed using EZR software (Saitama Medical Center, Jichi Medical University, Saitama, Japan).

## Results

### Expression of malignant or premalignant biomarkers by immunohistochemical staining in FVL and FVR boundary

**Fig. 4:**
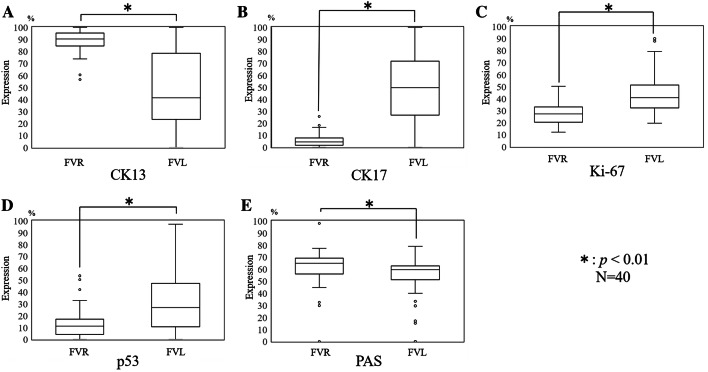
Expression rates of CK13, CK17, Ki67, p53, and PAS staining. The horizontal center line in each box indicates the median, and the vertical line indicates the expression rate. The upper edge of each box shows the third quartile deviation, and the lower edge shows the first quartile deviation

CK13 expression was consistently observed except in the basal cell layers. The CK13 expression rate was 91.6% in FVR and 41.4% in FVL (*p* < 0.01) (Fig. 4a).

CK17 expression was observed in the spinous cell layer and granular cell layer in FVL. In contrast, CK17 expression was rarely observed in any layers in FVR. The CK17 expression rate was 4.5% in FVR and 49.7% in FVL (*p* < 0.01) (Fig. 4b).

Ki-67 expression was scattered throughout the basal and spinous cell layers. The Ki-67 expression rate was 27.3% in FVR and 40.9% in FVL (*p* < 0.01) (Fig. 4c). The expression of p53 was chiefly found in cells of the basal layer and the lower or middle part of the spinous cell layer. The expression rate of p53 was 11.2% in FVR and 26.8% in FVL (*p* < 0.01) (Fig. 4d). The distribution patterns of Ki-67- and p53-positive cells in the FVL area were extremely similar, and reactive cells were found in not only the basal, but also the spinous cell layers.

### Comparison between FV device and iodine vital staining method

PAS-negative cells were observed in all layers of the epithelium in FVL. In contrast, PAS-positive cells were observed in only the spinous, granular, and keratinized layers in FVR. The expression rate of PAS staining was 59.5% in FVL and 64.9% in FVR (*p* < 0.01) (Fig. 4e). The FVL and IUS boundary were similar in many cases despite different mechanisms.

**Fig. 5:**
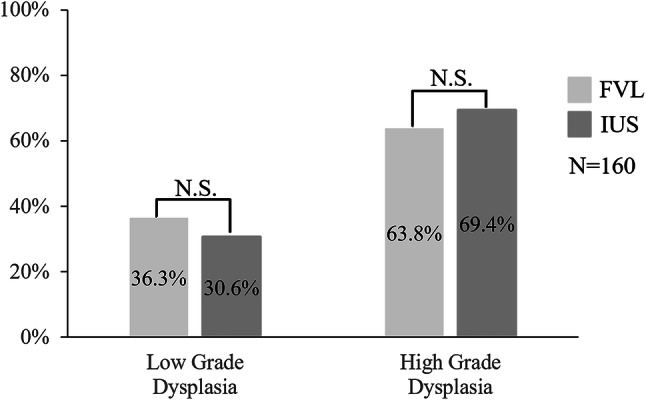
Description of lesion boundary. Various types of OED were observed at the lesion boundary between the area of fluorescence visualization loss (mild, 11.9%; moderate, 24.4%; severe, 63.8%) and iodine-unstained area (mild, 8.6%; moderate, 21.9%; severe, 69.4%). We classified high-grade dysplasia and low-grade dysplasia and found no significant differences (*p* = 0.34)

At the lesion boundaries, high-grade dysplasia was observed in 63.8% of cases at the FVL and in 69.4% of the IUS area. Low-grade dysplasia was observed in 36.3% of the FVL and in 30.6% of the IUS area. There were no significant histopathological differences between the FVL and IUS boundary (*p* = 0.34) (Fig. 5).

**Fig. 6:**
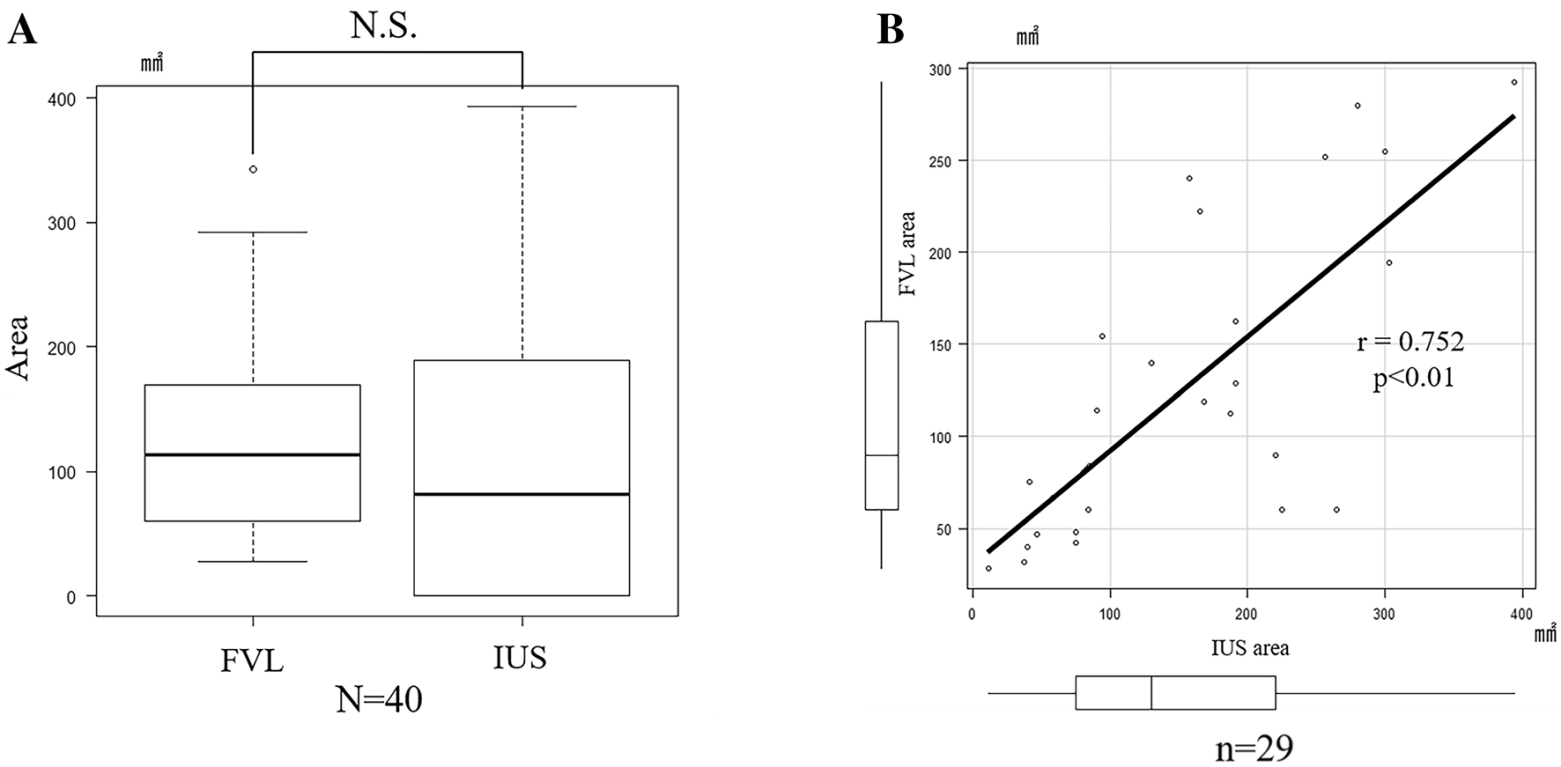
**a** Comparison of lesion delineation area. **b** Pearson’s product–moment correlation. 11 patients were excluded because the iodine-unstained (IUS) area did not appear in the iodine vital staining method. The data indicated a strong correlation between the fluorescence visualization loss (FVL) delineation area and the IUS delineation area (*p* < 0.01, 95% confidence interval = 0.532–0.877)

The effective rates for the FV device was 100% (40 cases). However, that for the iodine vital staining method was 72.5% (29 cases). The reason for the imperfection of iodine vital staining is that 9 of 11 cases could not be delineated, and the other 2 cases could not be examined because of iodine hypersensitivity. The lesion delineation rate was significantly higher with the FV device than iodine vital staining method (*p* < 0.01). However, the median lesion delineation area was 113.3 mm^2^ in FVL and 81.5 mm^2^ in the IUS area (*p* = 0.13) (Fig. 6a). The FVL and IUS areas demonstrated a strong correlation (*r* = 0.752, *p* < 0.01) (Fig. 6b).

## Discussion

The concept of “field cancerization” was introduced by Slaughter et al. [21] in 1953. The “field” refers to the abnormal tissue adjacent to the tumor, which develops in a multistep process of carcinogenesis. Furthermore, many recent studies have confirmed that this field has a high potential for malignant transformation with several types of oral epithelial dysplasia [4]. Morphologically, oral epithelial dysplasia develops into malignancy in 12.0–15.4% of cases [22, 23]. Additionally, some reports have stated that local recurrence occurs in 5.6–19.0% of T1–T2 early-stage OSCC [24–26]. Visualization of such an abnormal field with iodine vital staining was chosen because of its low cost, wide availability, and ease of use. McMahon et al. [6] reported that the use of Lugol’s iodine could reduce the incidence of dysplasia at the surgical margins in the resection of oral and oropharyngeal carcinoma. This direct FV method was more recently considered to have potential as a simple, noninvasive, repeatable screening device for early detection of oral premalignant lesions.

The purpose of this study was to prove that the FVL area is precancerous area histopathologically. Moll et al. [27] reported that several types of cytokeratin have great importance in immunohistochemical tumor diagnosis of carcinomas. CK13 is expressed in normal epithelium, and its expression tends to decrease in high-grade dysplasia [28]. Additionally, CK17 expression was higher in squamous cell carcinoma and lower in low-grade dysplasia and normal epithelium [29]. The expression of these cytokeratins indicates that they are accurate premalignant markers for evaluation of the abnormal field with malignant potential [17, 19, 30, 31]. In the present study, CK13 expression was significantly lower and CK17 expression was significantly higher at the FVL boundary than at the FVR. Thus, we found that the FVL area clinically reflects the concept of an abnormal field with malignant potential.

Ki-67 is a representative molecular marker of proliferating cells, and its labeling index correlates with the degree of high-grade dysplasia and severity of malignancy. Ohta et al. [32] reported that CK13 and Ki-67 expression was significantly different between iodine-stained and -unstained areas and that an abnormal field showing precancerous or cancerous features was detected at the IUS boundary. Moreover, p53 is a well-known tumor suppressor gene that introduces G1 arrest to allow the repair of DNA damage. Many studies have shown that p53 mutation occurs in the initial step of cancerization [17, 19, 33]. One study showed that the p53 expression rate was higher in oral epithelial dysplasia with high malignant potential than in carcinoma in situ or early-stage OSCC [34]. Taken together, these findings indicate that expression of Ki-67 and p53 in early-stage OSCC are important as initial signs of carcinogenesis [17, 19, 32, 33].

In the present study, we found that both Ki-67 and p53 were more highly expressed within abnormal fields under FV-guided surgery. Field cancerization based on the molecular abnormalities has shown that the presence of allelic loss at 3p, 9p, and 17p is associated with an increased cancer risk [4, 11]. A previous study showed an important step in the loss of heterozygosity analysis on each FVL boundary and a significant association of loss of heterozygosity at 3p and/or 9p based on mutations in TP53 [11]. These results may suggest that the FV device can delineate between the primary tumor and oral epithelial dysplasia with malignant potential based on field cancerization. Removal of the delineating area under FV-guided surgery may reduce the risk of a poor prognosis because of development a second primary tumor.

The present study also raises an important issue regarding the relative advantages of the iodine staining method and FV-guided surgery. The areas of normal epithelial tissue were PAS-positive because parakeratotic epithelial tissue contains a large amount of glycogen [18]. The PAS-negative area is well known to be equivalent to the IUS area [18]; likewise, most of the IUS area is correlated with high-grade dysplasia. In the present study, we found that all IUS areas consisted of oral epithelial dysplasia. Thus, the IUS areas that appeared in the 29 patients who underwent the performed iodine staining method were included in the resected specimens. Moreover, the FVL area was similar to the IUS area. The FVL boundary showed low- or high-grade dysplasia.

Several studies have shown that the pathological findings between the IUS and FVL areas might similarly consist of various types of dysplasia [35, 36]. However, the abnormal fields delineated by the FV- device are obviously obtained by different mechanisms than in the iodine vital staining method. For this reason, the FVL area was not always visually identical to the IUS area. The results of this study suggest that the FV- device is superior to the iodine staining method in aiding in the identification of the boundary of OED associated with OSCC prior to surgical resection. As described above, we recommend using not only iodine vital staining but also FV for detection of the surgical margin. The effective rate in iodine vital staining was about 70–80% [5, 7, 8, 35], although there is a difference depending on the iodine concentration. In this study, the results were almost the same. However, the iodine vital staining method exhibited imperfect delineating ability in nine cases and could not be performed because of iodine hypersensitivity in two cases. These two patients underwent only FV-guided surgery. FV-guided surgery may compensate for the shortcomings of the iodine vital staining method. Furthermore, the iodine vital staining method sometimes induced allergic reactions.

One of the limitations of complicated resection with iodine vital staining and FV is that only superficial lesions can be detected [11]. Advanced OSCC with deep invasion would cause no fluorescence alterations. However, the advantage of this device lies in the detection of early-stage and superficial of OSCC in clinically suspected cases that cannot be completely visualized by the naked eye. We hope that educational activities on early detection will be implemented to engage the medical community in the field of oral oncology and that the prognosis will be improved through further studies of FV-guided surgery. Accurate detection and differentiation of early-stage OSCC can have a significant effect on increasing the rate of early diagnosis. FV may be a beneficial tool in the evaluation of mucosal lesions when the situational context is taken into account. Our results showed a significant difference between normal and abnormal mucosa. In addition, analysis software that digitizes and graphs the difference in brightness between FVL and FVR is also being developed, and we believe that more accurate objective evaluation will be possible in the future, currently. The FV device appears to be a useful noninvasive aid in the management of potentially malignant lesions. In conclusion, we strongly suggest the performance of FV-guided surgery for accurate resection of primary tumors, including OED with malignant potential.
